# Three‐Year Durability of Radiofrequency Renal Denervation: SPYRAL HTN‐ON MED

**DOI:** 10.1161/JAHA.126.049081

**Published:** 2026-06-12

**Authors:** David E. Kandzari, Felix Mahfoud, Raymond R. Townsend, Kazuomi Kario, Michael A. Weber, Roland E. Schmieder, Konstantinos Tsioufis, Stuart Pocock, Guang Yang, Sandeep Brar, Michael Böhm

**Affiliations:** ^1^ Piedmont Heart Institute Atlanta GA USA; ^2^ Department of Cardiology, University Heart Center University Hospital Basel Switzerland; ^3^ Cardiovascular Research Institute Basel (CRIB), University Heart Center University Hospital Basel Basel Switzerland; ^4^ Perelman School of Medicine University of Pennsylvania Philadelphia PA USA; ^5^ Department of Cardiovascular Medicine Jichi Medical University School of Medicine Tochigi Japan; ^6^ SUNY Downstate College of Medicine New York NY USA; ^7^ University Hospital Erlangen Erlangen Germany; ^8^ National and Kapodistrian University of Athens, Hippocratio Hospital Athens Greece; ^9^ London School of Hygiene and Tropical Medicine London UK; ^10^ Medtronic Santa Rosa CA USA; ^11^ HOMICAREM (HOMburg Institute for CArdioREnalMetabolic Medicine), Saarland University Homburg Germany

**Keywords:** antihypertensive medications, hypertension, renal denervation, Hypertension, High Blood Pressure, Clinical Studies

## Abstract

**Background:**

The SPYRAL HTN‐ON MED (Global Clinical Study of Renal Denervation With the Symplicity Spyral Multielectrode Renal Denervation System in Patients With Uncontrolled Hypertension on Standard Medical Therapy) trial showed that radiofrequency renal denervation (RDN) using the Symplicity Spyral catheter yielded significant blood pressure (BP) reductions at 6 and 24 months compared with sham control in patients with hypertension on antihypertensive medications. In this prespecified analysis through the final follow‐up, we evaluate the durability of BP reductions, antihypertensive medication use, and safety through 3 years.

**Methods:**

SPYRAL HTN‐ON MED is a global, randomized, sham‐controlled trial enrolling patients with uncontrolled hypertension. Patients were prescribed 1 to 3 antihypertensive medications and randomized to RDN or a sham procedure. After 6 months, patients were unblinded, and sham patients could cross over to RDN. Crossover patients' values for BP and antihypertensive medications were imputed to 36 months using their last observation before crossover to RDN carried forward. Statistical analyses, including imputation, were exploratory and conducted on the intention‐to‐treat population.

**Results:**

After 6 months, 74% of sham patients crossed over to undergo RDN. Patients undergoing RDN had statistically significant 24‐hour ambulatory systolic BP reductions (treatment difference, –4.7 mm Hg; *P*=0.0028) and office systolic BP reductions (difference, –7.5 mm Hg; *P*=0.0002) compared with the sham control group through 36 months. At 36 months, the antihypertensive medication burden was balanced between groups (RDN, 5.2±5.0 versus 5.5±9.9; *P*=0.63). Crossover patients had significantly better office BP control after RDN. Clinical adverse events were rare.

**Conclusions:**

Compared with sham patients through 3 years, patients undergoing RDN had significantly greater 24‐hour ambulatory and office BP reductions, with a similar medication burden. RDN maintained a durable safety profile through 3 years.

**REGISTRATION:**

URL: https://clinicaltrials.gov/study; Unique identifier: NCT02439775.

**Nonstandard Abbreviations and Acronyms**
LOCF: last observation before crossover to renal denervation carried forward
RDN: renal denervation
SPYRAL AFFIRM: Global Study of RDN With the Symplicity Spyral Renal Denervation System in Subjects With Uncontrolled Hypertension
SPYRAL GEMINI: Global Pilot Study of Renal and Hepatic Combined DenervatIon in Subjects With Uncontrolled Hypertension With and Without High Cardiovascular Risk
SPYRAL HTN‐ON MED: Global Clinical Study of Renal Denervation With the Symplicity Spyral Multielectrode Renal Denervation System in Patients With Uncontrolled Hypertension on Standard Medical Therapy
SYMPLICITY HTN‐3: A Controlled Trial of Renal Denervation for Resistant Hypertension

**Clinical Perspective**

**What Is New?**

- In this prespecified analysis and final report from the SPYRAL HTN‐ON MED (Global Clinical Study of Renal Denervation With the Symplicity Spyral Multielectrode Renal Denervation System in Patients With Uncontrolled Hypertension on Standard Medical Therapy) trial, we evaluate the long‐term efficacy and safety of renal denervation at 3 years.

**What Are the Clinical Implications?**

- Compared with sham control, patients undergoing renal denervation had significantly greater long‐term systolic blood pressure reductions, with a similar antihypertensive medication burden.
- Renal denervation was safe, with no evidence of delayed safety complications, including in crossover patients.

Hypertension is a major global health problem affecting more than 1 billion adults.^1^ Uncontrolled hypertension is the leading risk factor for heart disease, heart failure, stroke, kidney disease, and peripheral vascular disease.^2^, ^3^, ^4^ Persistent blood pressure (BP) control is crucial to long‐term cardiovascular health, especially in those with other comorbidities.^5^, ^6^ Only about half of adults with hypertension are diagnosed, and only one‐fifth have their BP controlled.^7^ These observations exist despite the overwhelming evidence demonstrating the efficacy of lifestyle modifications and antihypertensive medications in reducing high BP.^8^, ^9^, ^10^, ^11^, ^12^, ^13^ Adherence to lifestyle modifications and prescribed medications remains poor, highlighting the need for an alternative therapeutic option for hypertension.

Catheter‐based renal denervation (RDN) targets the sympathetic nerves in the perivascular space of the kidney to reduce high BP.^14^, ^15^ This minimally invasive procedure was approved by multiple regulatory bodies, and is a guideline‐recommended therapy for uncontrolled hypertension in patients for whom medications and lifestyle interventions are insufficient to achieve BP control.^16^, ^17^, ^18^, ^19^, ^20^ The SPYRAL HTN‐ON MED (Global Clinical Study of Renal Denervation With the Symplicity Spyral Multielectrode Renal Denervation System in Patients With Uncontrolled Hypertension on Standard Medical Therapy) trial demonstrated significant reductions in office BP at 6 months and office and 24‐hour ambulatory BP at 2 years compared with a sham control procedure.^21^, ^22^ These results were despite a higher antihypertensive medication burden (considerate of medication number, class, and dose) in the sham control group. Multiple studies have demonstrated sustained, if not amplified, BP response to radiofrequency‐mediated RDN through long‐term follow‐up.^23^, ^24^ In this prespecified analysis of the SPYRAL HTN‐ON MED trial, we present the final follow‐up for all available patients, including those who crossed over, and we compare BP changes, antihypertensive medication changes, and safety results between treatment groups through 3 years of follow‐up.

## Methods

The data are proprietary of the funder but may be available upon reasonable request to the first author.

### Study Design and Patients

SPYRAL HTN‐ON MED is a global, randomized, blinded, sham‐controlled trial enrolling patients globally assessing the safety and efficacy of radiofrequency RDN using the Symplicity Spyral catheter (Medtronic plc, Galway, Ireland) for the treatment of hypertension in patients taking antihypertensive medications.^25^ Eligibility required patients to be aged 20 to 80 years with uncontrolled hypertension, defined as office systolic BP 150 to <180 mm Hg, office diastolic BP ≥90 mm Hg, and 24‐hour ambulatory systolic BP 140 to <170 mm Hg. Patients were required to be on a stable regimen of 1 to 3 antihypertensive medications through 6 months (primary end point).^21^ The initial 106 patients were randomized 1:1 to undergo RDN or the sham control procedure, after which patients were randomized 2:1. All patients provided written informed consent, and the protocol was approved by all review boards and local ethics committees. The study was designed in accordance with the Declaration of Helsinki.^26^

### Procedure

The RDN procedure using the Spyral catheter has been described previously.^21^, ^27^, ^28^, ^29^ The multielectrode RDN catheter and Symplicty G3 RDN radiofrequency generator (Medtronic plc) apply circumferential radiofrequency energy to modulate the sympathetic nerves surrounding the renal arteries and branch vessels 3 to 8 mm in diameter. Cases were performed by an experienced proceduralist and proctored on the basis of predetermined treatment plans. The sham procedure was limited to a renal angiogram requiring patients to remain on the procedure table for at least 20 minutes to preserve patient blinding.

### Follow‐Up

Follow‐ups were conducted at 1, 3, and 6 months and then yearly through 3 years. After witnessed pill intake, office BP was assessed by trial staff who were blinded to treatment allocation. Assessment of 24‐hour ambulatory BP was not conducted during the 1‐month follow‐up. Medication adherence was determined by urine and plasma testing. Medication changes were prohibited through 6 months unless prespecified criteria were met.^25^ Duplex ultrasound, computed tomography, or magnetic resonance imaging assessed renal artery anatomy in all patients at 6‐ and 12‐month follow‐up.

### Outcomes

Safety outcomes were assessed through 36 months, including a composite safety end point and each of its individual components: all‐cause death, end‐stage renal disease, significant embolic event resulting in end‐organ damage, renal artery perforation requiring intervention, renal artery dissection requiring intervention, vascular complications, hospitalization due to a hypertensive crisis not related to confirmed nonadherence with medications or the protocol, and new renal artery stenosis >70% confirmed by angiography determined by the angiographic core laboratory. Efficacy outcomes, including office and 24‐hour ambulatory BP, were assessed through 36 months.

### Crossover Patients, Procedure, and Follow‐Up

Sham control patients were permitted to cross over to RDN after the primary end point ascertainment (6 months) and unblinded without having to requalify per trial eligibility criteria. Crossover procedures were similarly performed by an experienced proceduralist and proctored. After crossing over, patients' follow‐up was an additional 3 years (1, 3, and 6 months; yearly).

### Statistical Analysis

All analyses were conducted according to the intention‐to‐treat principle on the basis of the patients' original randomization. Analyses of BP outcomes through 36 months were prespecified and presented in all patients with available data at the relevant follow‐up time point. Categorial variables are represented as percentages and counts, and comparisons between treatment groups use exact binomial tests. Continuous variables are reported as mean±SD. Treatment differences and medication differences between groups are compared using ANCOVA, adjusting for baseline measurements. As part of the primary analyses, all sham control patients who crossed over to undergo the RDN procedure before their final follow‐up had their last observation before crossover to RDN carried forward (LOCF; including BP, medications, estimated glomerular filtration rate, etc). Crossover LOCF imputation analyses were post hoc. A sensitivity analysis was also conducted without LOCF imputation. Adverse events were adjudicated by an independent clinical events committee. Sham patients who crossed over were censored for safety events at the time of crossover. Timelines for crossover patients’ BP measures began at the time of crossover. Statistical analyses were conducted using SAS for Windows 9.4 (SAS Institute, Cary, NC).

### Role of the Funding Source

Medtronic plc funded the SPYRAL HTN‐ON MED study. The executive committee designed the protocol and identified suitable clinical sites to conduct the study in collaboration with the funder. The funder was responsible for data collection, monitoring, and analysis. The lead author wrote the manuscript with contributions from co‐authors and copy‐editing assistance from the funder. Medtronic assisted in figure and table generation and manuscript formatting. All authors had full access to the data and were responsible for the decision to submit for publication. The data from the Spyral HTN‐ON MED trial are proprietary under Medtronic and are not publicly available.

## Results

### Patient Characteristics

**Figure 1:**
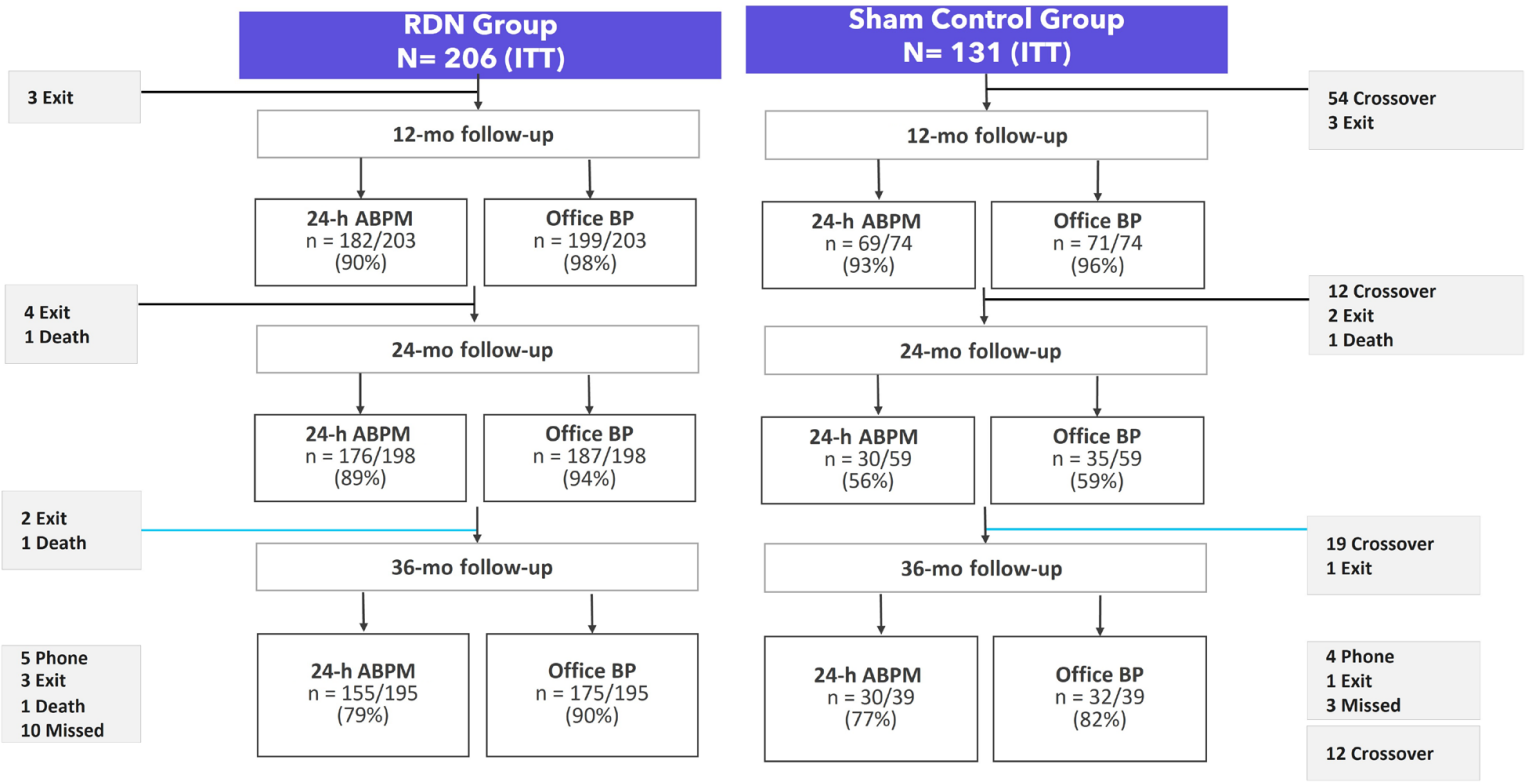
SPYRAL HTN‐ON MED patient flowchart through 36 months. After the primary end point ascertainment at 6 months, sham control patients were allowed to cross over to undergo the RDN procedure without having to requalify per the original enrolment criteria. Percentages show proportion of patients with observed outcomes. Follow‐up by phone was permitted for sham control patients after the primary end point. ABPM indicates 24‐hour ambulatory blood pressure measures; ITT, intention‐to‐treat; and SPYRAL HTN‐ON MED, Global Clinical Study of Renal Denervation With the Symplicity Spyral Multielectrode Renal Denervation System in Patients With Uncontrolled Hypertension on Standard Medical Therapy.

From July 22, 2015, to February 15, 2022, 1780 patients were enrolled from 56 international clinical centers. Three‐hundred thirty‐seven patients were eligible per trial criteria (Methods) and randomly assigned to the RDN procedure (n=206) or the sham control procedure (n=131; Figure 1). Eleven patients from the RDN group and 5 control patients exited the trial before the 3‐year follow‐up. Sham control patients were permitted to cross over to undergo the RDN procedure after the primary end point ascertainment (6‐month follow‐up), without having to requalify for eligibility per the original trial criteria. Ninety‐seven control patients (74%) crossed over, with a plurality (n=54) crossing between the 6‐ and 12‐month follow‐up visit, but some patients (n=12) crossed over after the 3‐year follow‐up. The mean time for crossover after randomization was 444±324 days (14.8 months [median, 252 days]). Crossover patients were censored at the time of their RDN procedure, and their most recent measures were imputed by their LOCF through 36‐month follow‐up as part (73% [n=85]) of the sham control group (Methods). At the 3‐year time point, 5 patients undergoing RDN and 4 sham control patients were followed‐up remotely via phone call and thus had no BP measures available, and 10 patients undergoing RDN and 3 control patients missed their final follow‐up visit (Figure 1). The baseline characteristics were mostly similar between groups, including baseline BP (Table).^21^, ^23^ No patient in the RDN or crossover group received a repeat ablation during the 3‐year follow‐up period.

**Table 1:** Baseline Patient Characteristics

Characteristic	RDN (n=206)	Sham control (n=131)
24‐h ambulatory systolic BP, mm Hg	149.6±7.0	149.3±7.0
24‐h ambulatory diastolic BP, mm Hg	96.6±7.6	95.7±7.7
Office systolic BP, mm Hg	163.0±7.7	163.1±7.9
Office diastolic BP, mm Hg	101.2±7.0	101.5±7.3
Age, y	55.2±9.0	54.6±9.4
Male sex	167 (81.1)	103 (78.6)
Body mass index, kg/m^2^	31.4±6.0	32.1±5.2
Estimated glomerular filtration rate, mL/min per 1.73 m^2^	81.9±16.8	81.9±17.2
Length of hypertension diagnosis, >5 y	144 (69.9)	107 (81.7)
Race and ethnicity, Black American	35 (17.0)	25 (19.1)
Type 2 diabetes	22 (10.7)	23 (17.6)
Current smoker	32 (15.5)	21 (16.0)
Obstructive sleep apnea	23 (11.2)	23 (17.6)
History of coronary artery disease	11 (5.3)	9 (6.9)

Values are reported as mean±SD or n (%). BP indicates blood pressure; and RDN, renal denervation.

### Antihypertensive Medication Changes

**Figure 2:**
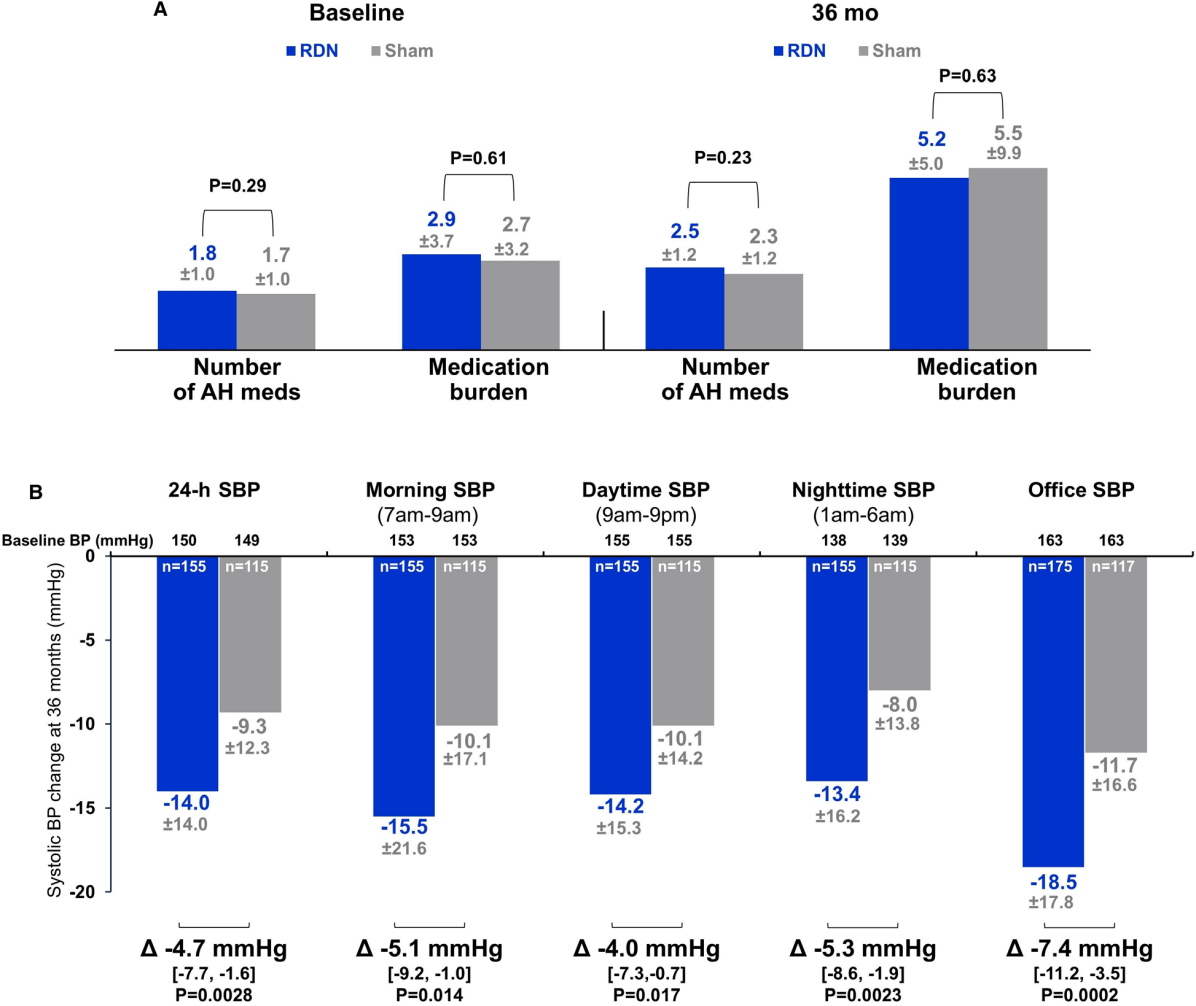
Antihypertensive medication intake and systolic BP changes in RDN and sham control groups through 36 months. The number of antihypertensive medications and the medication burden (accounting for number, class, and dose)±SD are plotted in (**A**) at baseline and 36 months. The RDN group is shown in blue, and the sham control group is shown in gray. The plotted data are based on drug testing information if available; otherwise, prescribed information is used. In (**B**), the 24‐hour ambulatory, morning (7 am to 9 am), daytime (9 am to 9 pm), nighttime (1 am to 6 am), and office‐visit systolic BP changes ±SD from baseline are plotted for the RDN and sham control groups. Comparisons of medication intake and BP changes were conducted by ANCOVA. For BP measures, comparisons were adjusted for baseline BP, with 95% CIs in brackets. BP indicates blood pressure; and RDN, renal denervation.

The number of antihypertensive medications based on available drug testing results was balanced between groups at baseline.^21^ If drug‐testing data were not available, prescribed information was used. The number of antihypertensive medications remained balanced through 36 months between the RDN and sham control groups (2.5±1.2 versus 2.3±1.2; *P*=0.23; Figure 2). Likewise, the antihypertensive medication burden, accounting for the medication number, class, and dosage, was also similar between groups at 36 months (5.2±5.0 versus 5.5±9.9; *P*=0.63).

### BP Changes

**Figure 3:**
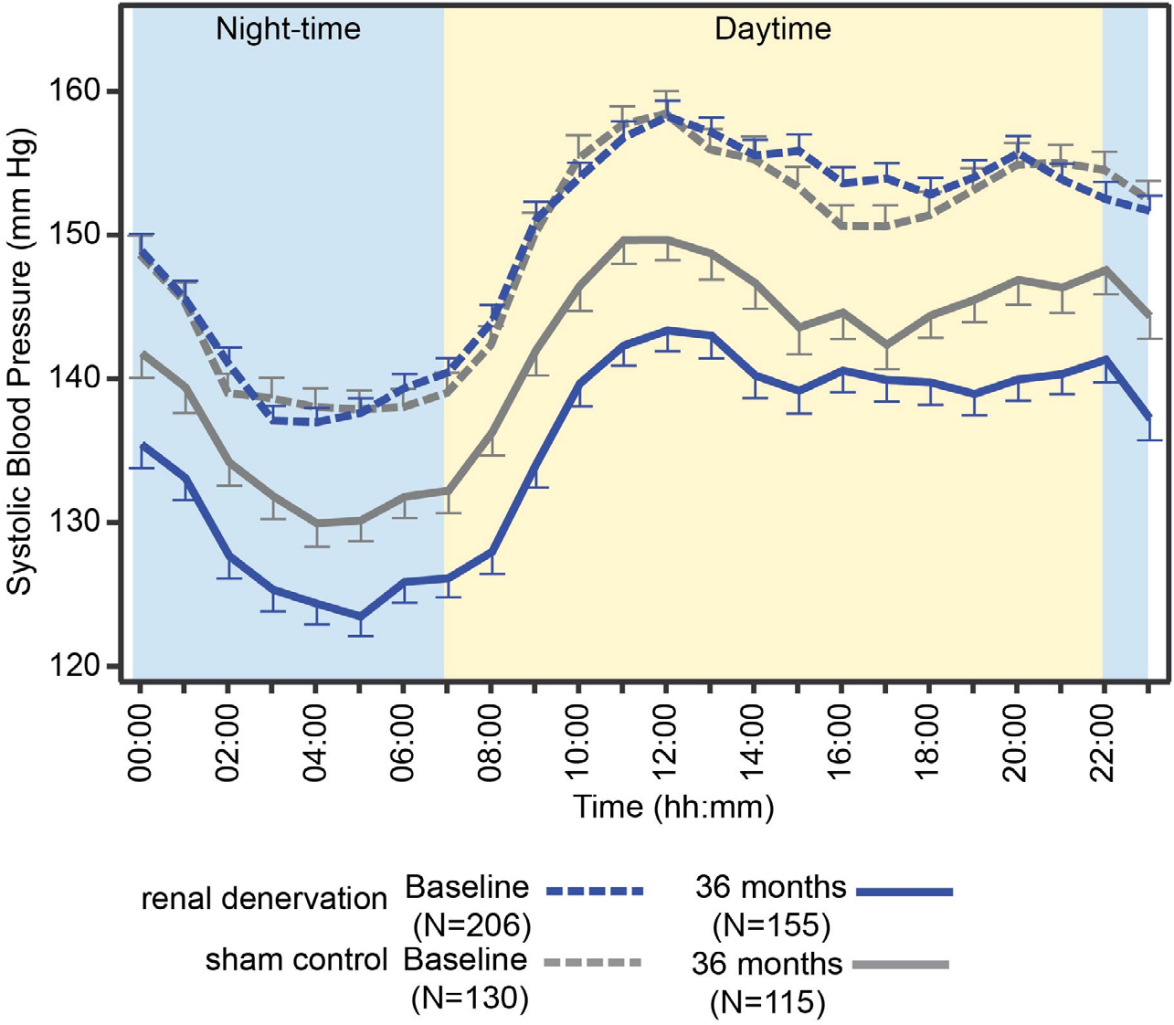
Hourly ambulatory blood pressure at baseline and 36 months between RDN and sham control groups. Hourly ambulatory systolic BPs at baseline and 36 months in the RDN (blue) and sham control groups (gray). BP indicates blood pressure; and RDN, renal denervation.

**Figure 4:**
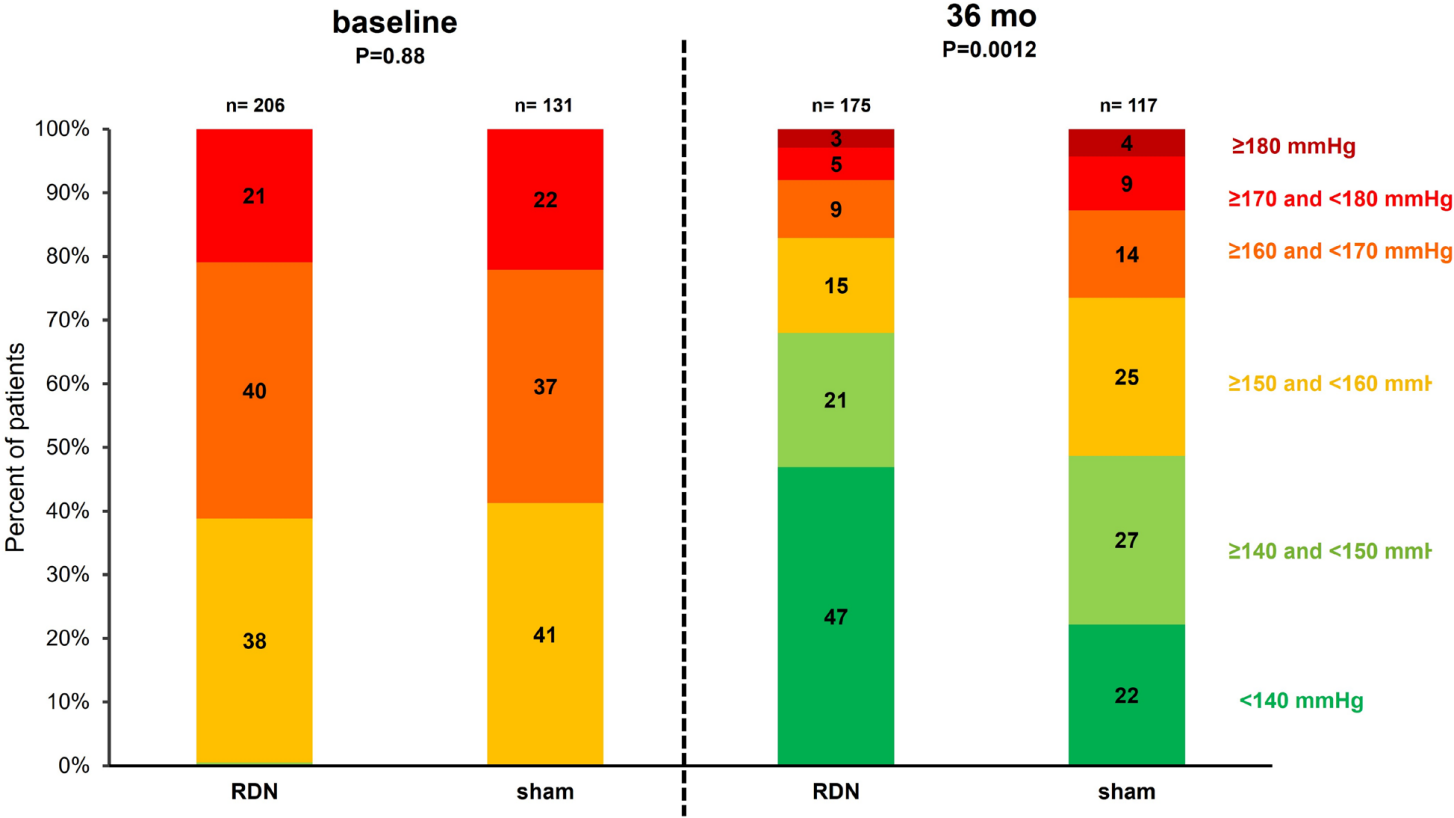
Distribution of office systolic BP in the RDN group versus sham control group through 36 months. The proportion of patients in the indicated office systolic BP ranges at baseline and 36 months. Distribution comparisons between RDN and sham control groups were conducted via Fisher's exact tests. BP indicates blood pressure; and RDN, renal denervation.

At 36 months, patients undergoing RDN compared with sham control patients had significantly greater reductions in 24‐hour ambulatory systolic BP (−14.0±14.0 mm Hg versus −9.3±12.3 mm Hg; ANCOVA‐adjusted treatment difference, −4.7 mm Hg [95% CI, −7.7 to −1.6 mm Hg]; *P*=0.0028) and office systolic BP (−18.5±17.8 mm Hg versus −11.7±16.6 mm Hg; ANCOVA‐adjusted treatment difference, −7.4 mm Hg [95% CI, −11.2 to −3.5 mm Hg]; *P*=0.0002) (Figure 2). Ambulatory systolic BP reductions were consistently in favor of patients undergoing RDN across the 24‐hour circadian cycle (Figure 3), including during daytime and nighttime (Figure 2). Moreover, the RDN group had a significant improvement in BP control relative to the sham control group at 36 months (Figure 4). Comparison of diastolic BP changes at 36 months showed similar trends (Figures S1 and S2).

We performed several sensitivity analyses (Figure S3), including a comparison between patients undergoing RDN and the sham control patients who did not cross over without imputation (Table S1). There was no significant BP treatment difference between groups at 36 months without imputation; however, patients undergoing RDN took significantly fewer medications (2.5±1.2 versus 3.0±1.6; *P*=0.019) and had a significantly lower medication burden (5.2±5.0 versus 10.4±16.5; *P*=0.0005) at 36 months (Table S2). An additional post hoc sensitivity analysis in which all missing measures for both the RDN and sham control groups (including those lost to follow‐up) were imputed to 36 months showed office and 24‐hour ambulatory systolic BP treatment differences in favor of RDN (Figure S4). Office and 24‐hour ambulatory systolic BP reductions at 36 months across various prespecified subgroups including sex, age, body mass index, diabetes status, Black American status, and baseline BP are provided in Figure S5. Finally, a comparison of outcomes in patients with resistant hypertension (those taking 3 antihypertensive medications at baseline) is provided in Figure S6.

### Safety Outcomes

Overall, adverse events were rare (Table S3), and through 36 months, the rate of the composite safety end point was similar between RDN and sham control groups (3.3% versus 2.7%). At 36 months, the change in estimated glomerular filtration rate was −2.9±12.0 mL/min per 1.73 m^2^ for the RDN group and −2.4±9.3 mL/min per 1.73 m^2^ for the sham control group (*P*=0.68). There were no instances of new renal artery stenosis >70% or renal artery stent implant in either cohort.

### Crossover Patient Outcomes

**Figure 5:**
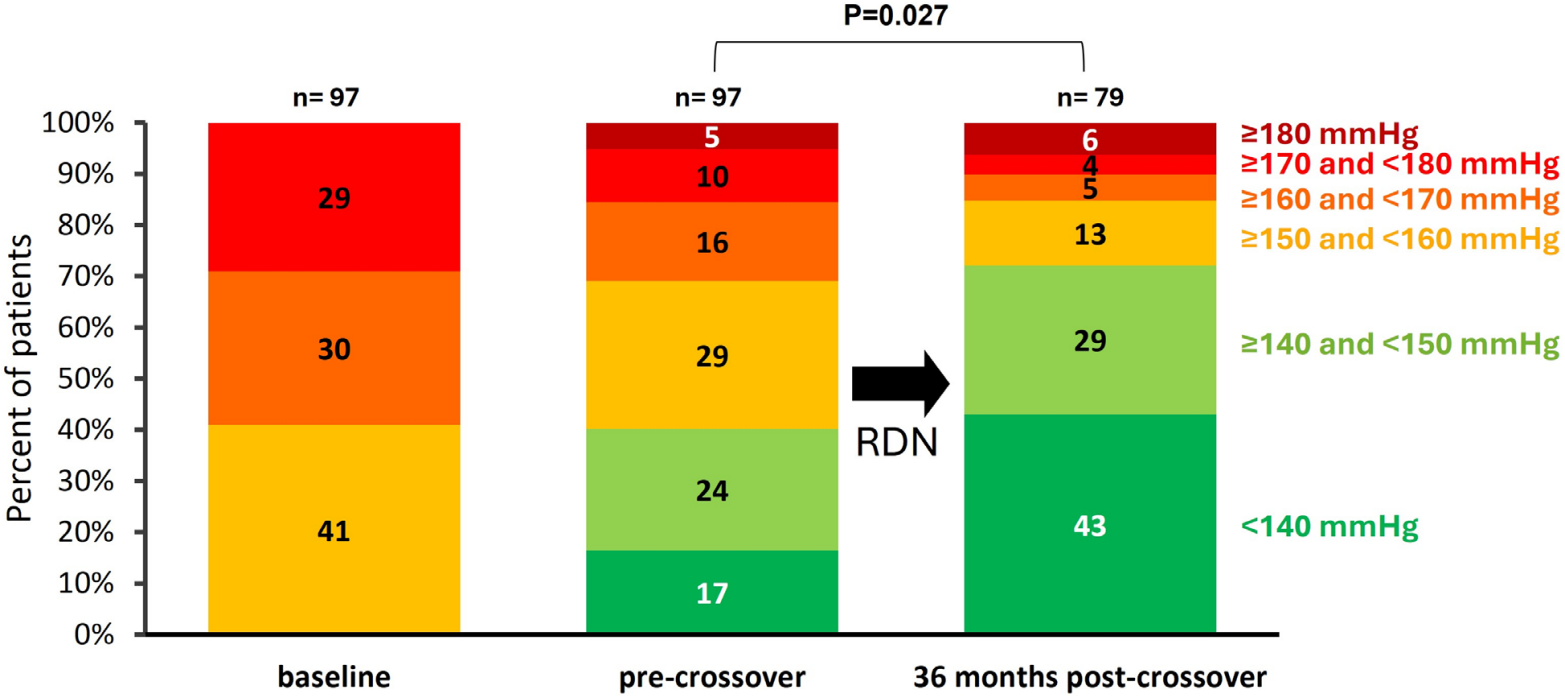
Distribution of office systolic BP among crossover patients before and after the RDN procedure. The proportion of patients in the indicated office systolic BP ranges at baseline, 6 months after randomization, the last measure before crossover, and 36 months after crossover. Distribution comparison between time points was conducted via McNemar–Bowker test. BP indicates blood pressure; and RDN, renal denervation.

The distributions of office systolic BP were also assessed among sham control patients who crossed over to undergo RDN (Figure 5). Among crossover patients (Table S4), 83% remained uncontrolled (office systolic BP ≥140 mm Hg) before crossing over (antihypertensive medication burden, 4.0±5.3). However, 36 months after RDN, many crossover patients (43%) had an office systolic BP <140 mm Hg. The medication burden 36 months after crossing over was 5.0±4.9, which was not a significant increase (0.8±5.2; *P*=0.16) from the precrossover medication burden.

## Discussion

In this 3‐year report of the SPYRAL HTN‐ON MED trial, we present the final follow‐up of the study including crossover patient outcomes. There were significant reductions in both 24‐hour ambulatory and office systolic and diastolic BP at 36 months were observed after RDN as compared with sham control. This was despite a similar medication burden between groups. Treatment differences were observed throughout the 24‐hour period including the morning, daytime, and nighttime. Comparison of the distribution of patients' office systolic BP through 36 months also showed a significantly greater proportion of patients undergoing RDN had an office systolic BP under the trial eligibility criterion of 150 mm Hg compared with control patients. Moreover, sham control patients who crossed over to RDN therapy following the primary end point also demonstrated a significant improvement in office systolic BP, similar to the original RDN group at 3 years.

An inherent challenge for any interventional trial permitting crossover is the attenuation of available long‐term data from the sham control group, especially when crossover patients are not randomly selected from the sham control population.^30^ In such instances, data imputation by LOCF is an appropriate and accepted statistical method to allow comparison between the treatment and sham groups.^31^, ^32^, ^33^ Longitudinal follow‐up of both drug and device trials in hypertension indicate that BP control worsens in the control group beyond the primary end point,^13^, ^30^ supporting LOCF as a conservative estimate. Importantly, an additional analysis comparing RDN versus sham control patients in which all missing patient data from both RDN and sham arms were imputed showed similar results to the primary analysis. Without imputation, 36‐month BP changes were more comparable between groups; however, this was balanced by the significantly higher antihypertensive medication burden in the sham control group. In fact, sham patients' medication burden was double that of the RDN group.

Notably, significant BP reductions were observed among sham patients who crossed over to RDN after the primary 6‐month follow‐up period. In this group, BP improved somewhat before crossover due to medication escalation, parallel to the overall sham cohort at 36 months. However, following RDN, the crossover group experienced further BP reductions similar to those achieved by patients undergoing RDN and with fewer medications than sham patients who did not cross over. These findings highlight the complementary benefit of RDN to medications for meaningful, incremental BP reduction.

The present results directly address the unmet need for long‐term comparative safety and efficacy data for the RDN procedure highlighted in recently published guidelines^17^, ^18^, ^19^ and position papers^20^, ^34^ and build upon a growing body of evidence supporting the durable efficacy and safety of RDN. Previous reports from the Spyral HTN‐ON MED study pilot cohort,^31^ 2‐year data from the full cohort,^22^ and 3‐year data from the SYMPLICITY HTN‐3 trial (A Controlled Trial of Renal Denervation for Resistant Hypertension) all consistently show sustained BP reduction and excellent procedural safety.^30^ A recent mixed‐model regression analysis incorporating all available long‐term, patient‐level information from the Symplicity RDN program, including over 4000 patients and 12 000 patient‐years of data, found sustained reductions in BP with radiofrequency RDN that gradually increased in magnitude through 3 years.^23^ Moreover, the mixed‐model analysis evaluated whether certain patient characteristics, including demographics, kidney function, and medication classes, among others, were associated with a greater response to RDN. However, the study found that only higher baseline BP was consistently associated with greater BP reductions.

The durable efficacy with RDN does not appear to be specific to the trial setting, as numerous studies have found sustained, long‐term reductions in BP even beyond 3 years and as long as 10 years.^24^, ^35^, ^36^ These findings cannot be explained by an increase in medications alone and may suggest that RDN is a “1‐time” procedure. Likewise, clinicians should consider the timing and dynamic response to the RDN procedure and counsel therapy candidates that the full benefit of RDN may take months, perhaps beyond 1 year, to become fully apparent. It is critical for patients to continue to adhere to lifestyle modifications and prescribed antihypertensive drug therapy following the RDN procedure. The mechanisms of the increased efficacy over time remains incompletely understood^23^ but may include attenuation of the renin–angiotensin–aldosterone system, reduced peripheral vascular resistance, and interruption or resetting baroreflex.^37^, ^38^, ^39^, ^40^

The Symplicity SPYRAL HTN randomized, sham‐controlled trial program offers multiple lessons for future device‐based therapy studies in hypertension. Both the SPYRAL HTN‐OFF MED and ON MED trials used a Bayesian design, that enabled a reduction in the number of patients required to achieve statistically powered end points and allowed adaptive adjustments and interim analyses to improve the trials' efficiency. Another lesson from the SPYRAL HTN program is how crossovers can potentially confound long‐term comparisons. In an effort to accelerate patient recruitment, sham control patients were permitted to cross over after the primary end point without having to requalify on the basis of original trial enrollment criteria. However, permitting crossover necessitates imputation methods for those patients to have any meaningful comparison between treatment arms. Potentially exacerbating this problem, a sizeable fraction of sham control patients achieved controlled BP by uptitrating antihypertensive medications,^41^ yet opted to cross over anyway. Patient preference for an interventional procedure for even incremental BP control, despite potential risks, has been previously documented.^42^, ^43^

This final report concludes the SPYRAL HTN randomized trial program and marks a critical new milestone for RDN as a therapy. Multiple guidelines now recommend RDN as the third therapeutic pillar, along with lifestyle modifications and antihypertensive medications, for patients with uncontrolled hypertension.^16^, ^17^, ^18^, ^19^, ^20^ The ongoing SPYRAL AFFIRM (Global Study of RDN With the Symplicity Spyral Renal Denervation System in Subjects With Uncontrolled Hypertension; NCT05198674) trial is designed to confirm the safety and efficacy of RDN reported in the ON MED trial, but will also evaluate the therapy in higher risk populations not included in the sham‐controlled trials, such as those with chronic kidney disease. There are several other studies investigating RDN's utility in treating other disease states related to sympathetic nervous system overactivity, such as heart failure, atrial and ventricular arrythmia, sleep apnea, and chronic kidney disease.^44^, ^45^, ^46^, ^47^, ^48^ The ongoing SPYRAL GEMINI (Global Pilot Study of Renal and Hepatic Combined DenervatIon in Subjects With Uncontrolled Hypertension With and Without High Cardiovascular Risk; NCT06907147) trial will evaluate the safety of multiorgan denervation (hepatic and renal) using the Spyral catheter and assess the efficacy in the treatment of hypertension.^49^, ^50^

### Limitations

There are several important limitations to this study. After the primary end point at 6 months after randomization, patients and staff were no longer blinded, and the protocol allowed clinicians to alter medications. Continued clinical surveillance may have motivated clinicians to escalate medications to improve BP control.^41^ Furthermore, patients were aware of the trial end points and could modify medication intake. Drug adherence testing partially rectifies this limitation; however, medication dosage could not be determined via drug testing. Patient adherence to lifestyle interventions was not captured. As discussed previously, assessment of the intergroup difference between RDN and sham is impacted since relatively few sham control patients have 36‐month BP measures due to crossover. Moreover, imputation by LOCF assumes there is no temporal trend if patients did not cross over. Nonetheless, a majority of sham control patients crossed over to undergo RDN and did not do so at random. In fact, most crossover patients would have no longer been eligible as per the original trial enrollment criteria at the time of their last observation before the RDN procedure. This may bias imputation analyses using LOCF in favor of sham, since it is unknown whether these patients would maintain a relatively low BP without crossing over. While analysis with imputation demonstrated a significant treatment effect in favor of RDN, analysis without imputation demonstrates that patients undergoing RDN had a significantly lower medication burden. Finally, some BP measures were not available from several sham control patients after 12 months during the pilot phase due to the initial trial design.^25^

## Conclusions

In this final, predefined follow‐up from the randomized SPYRAL HTN‐ON MED trial, RDN demonstrated clinically meaningful and significant office and 24‐hour ambulatory BP reductions compared with sham. There was no evidence of delayed safety complications after RDN. Sham group patients who eventually crossed over to undergo RDN had BP reductions and antihypertensive medication burdens similar to the RDN group at 3 years. These results add to the growing body of evidence of the durable efficacy and safety of RDN as the third therapeutic pillar in the treatment of uncontrolled hypertension.

## Sources of Funding

This work was funded by Medtronic Plc.

## Disclosures

Dr Kandzari discloses receives institutional research/grant support from Biotronik, Boston Scientific, Orbus Neich, Teleflex, Medtronic, and Ablative Solutions; he also receives personal consulting honoraria from Ablative Solutions, Medtronic, and HyperQure. Dr Mahfoud has been supported by Deutsche Forschungsgemeinschaft (SFB TRR219, Project ID: 322900939), and Deutsche Herzstiftung. Saarland University has received scientific support from Ablative Solutions, Medtronic and ReCor Medical. Until May 2024, Dr Mahfoud has received speaker honoraria/consulting fees from Ablative Solutions, Astra‐Zeneca, Inari, Medtronic, Merck, Novartis, Philips, and ReCor Medical. Dr Townsend is a consultant for Medtronic, Axio, Regeneron, Bard, OBIO, Corcept, and AstraZeneca; and has received royalties from UpToDate. Dr Kario receives personal fees from Medtronic, receives grants from A&D Company, JIMRO, Omron Healthcare, CureApp, Terumo, and Fukuda Denshi; receives honoraria from Otsuka Pharmaceuticals and Omron Healthcare; and participates on the advisory board of Fukuda Denshi outside the submitted work. Dr Weber has received consulting fees from Medtronic, ReCor, Ablative Solutions, Johnson & Johnson, and Urovant. Dr Schmieder has received speaker and consulting honoraria from Medtronic, Recor, and Ablative Solutions. Research grants have been given to his Institution from Medtronic, Recor, Sonivie, and Ablative Solutions. Dr Tsioufis reports institutional research/grant support from Medtronic and ReCor Medical and personal consulting honoraria from Astra‐Zeneca, Bayer, Boehringer Ingelheim, Medtronic, ReCor Medical, SERVIER, WinMedica, and ELPEN. Dr Pocock reports personal fees from Medtronic, Edwards LifeSciences, and Boston Scientific. G. Yang and Dr Brar are employees of Medtronic. Dr Böhm is supported by the Deutsche Forschungsgemeinshaft (SFB TTR219) and receives personal fees from Abbott, Amgen, AstraZeneca, Bayer, Boehringer Ingelheim, Cytokinetics, Edwards, Medtronic, Novartis, ReCor Medical, Servier, and Vifor.

## Supporting information

Tables S1–S4
Figures S1–S6

STROBE Statement
