# Supplementary material for: Three‐Year Durability of Radiofrequency Renal Denervation: SPYRAL HTN‐ON MED
Source: J Am Heart Assoc. 2026 Jun 12;15(12):e049081. doi: 10.1161/JAHA.126.049081 (PMC13323591; doi:10.1161/JAHA.126.049081)
Supplement: Supplementary file 1 — Tables S1–S4 Figures S1–S6 [file JAH3-15-e049081-s002.pdf]

Supplemental Material

Table S1. Office and 24-h ambulatory blood pressure changes through 36 months without imputation.

mean±SD (n)	24-h ambulatory systolic BP change			Office systolic BP change		
	RDN	Sham	p-value	RDN	Sham	p-value
12 months	-9.6±12.2 (n=182)	-9.1±11.5 (n=69)	0.71	-14.3±16.1 (n=199)	-11.6±15.3 (n=71)	0.15
24 months	-12.1±15.3 (n=176)	-7.0±13.1 (n=33)	0.039	-17.4±16.1 (n=187)	-9.0±19.4 (n=35)	0.0034
36 months	-14.0±14.0 (n=155)	-10.7±14.1 (n=30)	0.16	-18.5±17.8 (n=175)	-18.7±16.7 (n=32)	0.92
	24-h ambulatory diastolic BP change			Office diastolic BP change		
12 months	-6.8±8.5 (n=182)	-6.4±8.3 (n=69)	0.71	-7.4±10.4 (n=199)	-7.0±9.0 (n=71)	0.53
24 months	-8.6±10.4 (n=176)	-5.3±9.0 (n=33)	0.057	-9.2±10.4 (n=187)	-4.8±10.5 (n=35)	0.020
36 months	-10.1±8.8 (n=155)	-6.7±8.3 (n=30)	0.10	-9.2±10.7 (n=175)	-11.4±10.3 (n=32)	0.22

Comparisons are ANCOVA adjusted for differences in baseline measures.

Table S2. Antihypertensive medications and medication burden through 36 months without imputations

mean±SD (n)	Number of medications			Medication burden		
	RDN	Sham	p-value	RDN	Sham	p-value
Baseline	1.8±1.0 (n=206)	1.7±1.0 (n=131)	0.29	2.9±3.7 (n=206)	2.7±3.2 (n=131)	0.61
6 months	1.9±1.0 (n=205)	1.9±0.9 (n=130)	0.058	3.1±3.8 (n=205)	3.2±3.1 (n=130)	0.38
12 months	2.2±0.9 (n=200)	2.5±1.1 (n=74)	0.0039	3.9±3.1 (n=200)	5.5±5.2 (n=74)	0.0003
24 months	2.4±1.2 (n=194)	2.7±1.2 (n=59)	0.048	4.8±4.7 (n=194)	6.1±5.6 (n=59)	0.056
36 months	2.5±1.2 (n=179)	3.0±1.6 (n=36)	0.019	5.2±5.0 (n=179)	10.4±16.5 (n=36)	0.0005

Baseline comparisons are from t-tests. Follow-up comparisons are ANCOVA adjusted for baseline measures.

Table S3. Safety events through 36 months

% (n)	RDN (n=184)	Sham Control (n=113)
Composite Safety Endpoint[1]	3.3% (6)	2.7% (3)
Death	1.1% (2)	0.9% (1)
New MI	1.1% (2)	0.0% (0)
New Stroke	0.5% (1)	0.9% (1)
Major Bleeding (Thrombolysis in Myocardial Infarction)	0.0% (0)	0.0% (0)
Significant embolic event resulting in end-organ damage	0.5% (1)	0.0% (0)
Vascular complications requiring surgical repair, interventional procedure, thrombin injection, or blood transfusion	1.1% (2)	1.8% (2)
Hospitalization for hypertensive crisis/emergency	0.5% (1)	0.0% (0)
Renal artery re-intervention	0.0% (0)	0.0% (0)
New Renal Artery Stenosis > 70%	0.0% (0)	0.0% (0)

Adverse event rates through 36 months. [1] Composite safety endpoint is all cause mortality, end-stage renal disease, significant embolic event resulting in end-organ damage, renal artery perforation requiring intervention, renal artery dissection requiring intervention, vascular complications, hospitalization due to a hypertensive crisis not related to confirmed non-adherence with medications and/or the protocol, and new renal artery stenosis >70% confirmed by angiography determined by the angiographic core laboratory.

Table S4. Baseline characteristics among crossover and non-crossover sham control patients

Mean±SD or % (n)	Crossovers (n=97)	Non-crossovers (n=34)
24-h ambulatory systolic BP, mm Hg	149±7	149±7
24-h ambulatory diastolic BP, mmHg	96±8	96±8
Office systolic BP, mm Hg	164±9	162±5
Office diastolic BP, mm Hg	102±7	100±8
Age, y	54.0±9.1	56.4±10.4
Male	76% (74)	85% (29)
BMI, kg/m^2	32.2±5.2	32.1±5.1
eGFR, mL/min/1.73 m^2	81.8±17.1	82.1±17.8
Length of hypertension diagnosis, >5y	86% (83)	71% (24)
Black Americans (race)	19% (18)	21% (7)
Type 2 diabetes mellitus	14% (14)	27% (9)
Current smoker	11% (11)	29% (10)
Obstructive sleep apnea	21% (20)	9% (3)
History of coronary artery disease	8% (8)	3% (1)

BP indicates blood pressure; BMI, body mass index; BP, blood pressure; eGFR, estimated glomerular filtration rate; and RDN, renal denervation.

Figure S1. 24-hour ambulatory, morning, daytime, nighttime, and office diastolic blood pressure changes from 36 months

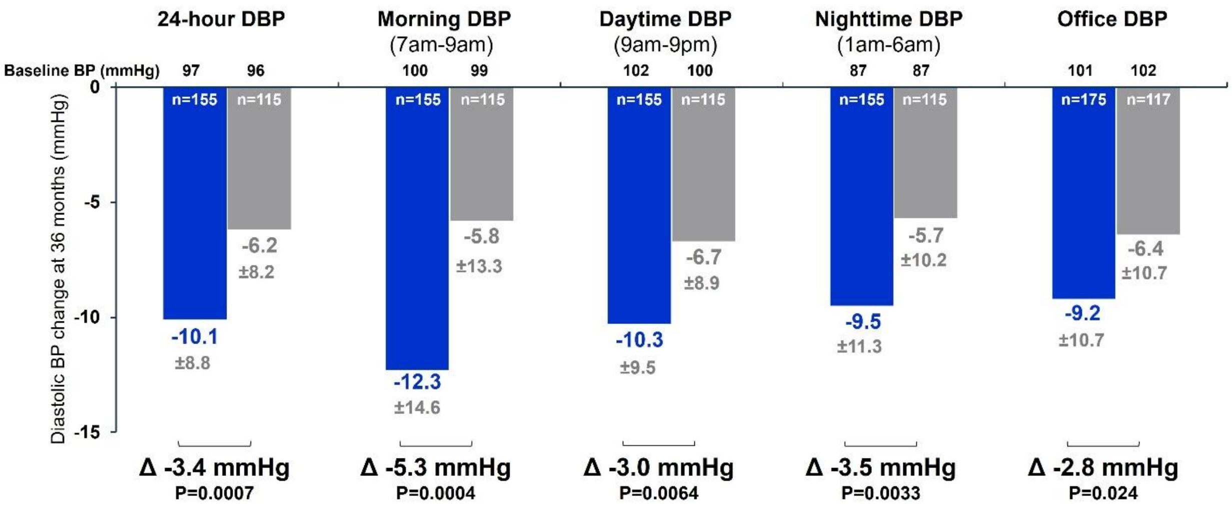

24-hour ambulatory, morning (7am-9am), daytime (9am-9pm), nighttime (1am-6am), and office-visit diastolic blood pressure (BP) changes from baseline are plotted for RDN (blue) and sham control groups (gray) ± the standard deviation. Comparisons were adjusted for baseline BP.

Figure S2. Hourly diastolic blood pressure at baseline and 36 months

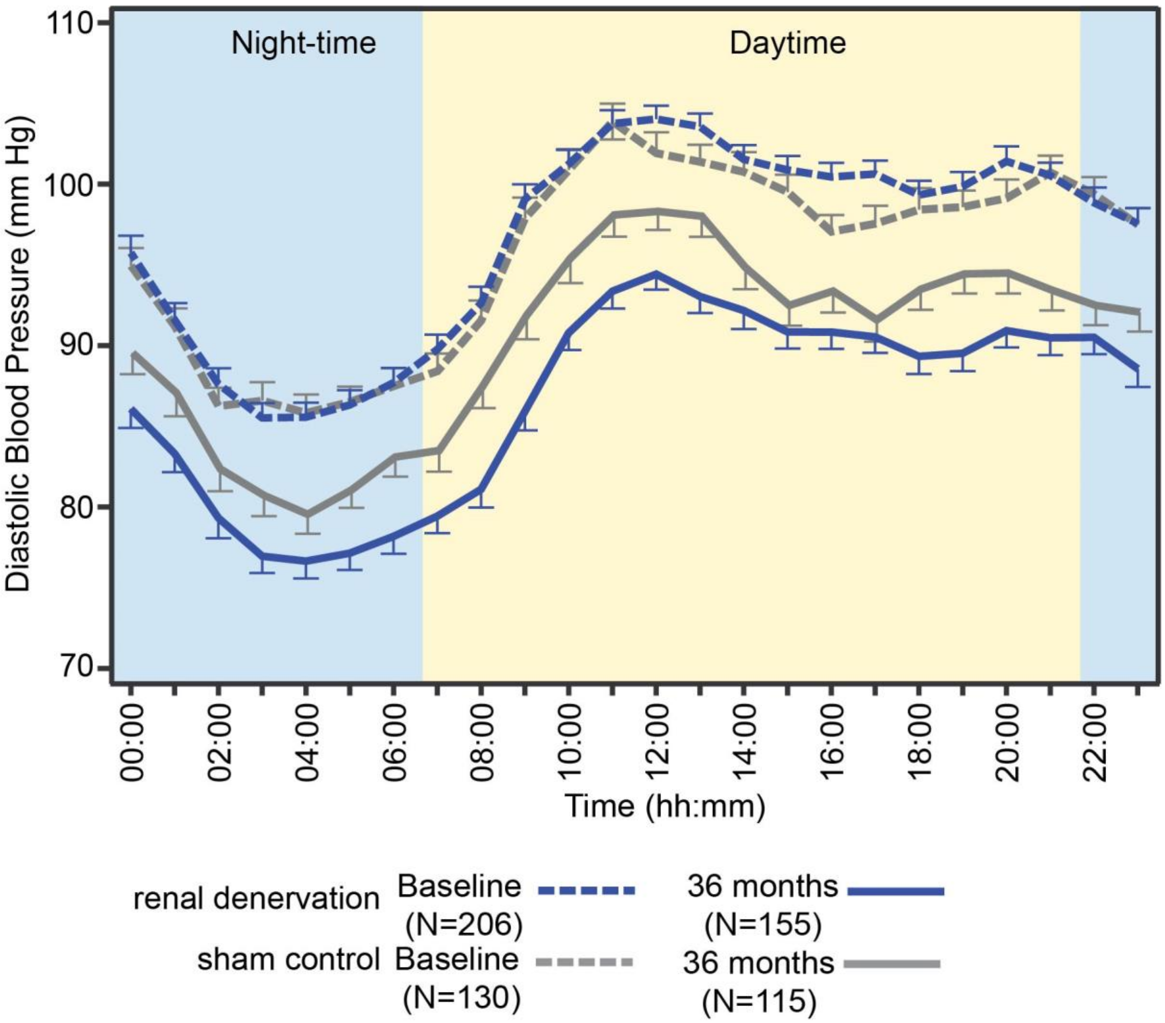

Hourly ambulatory diastolic BPs at baseline and 36 months in the RDN (blue) and sham control groups (gray).

Figure S3. Summary of sensitivity analyses

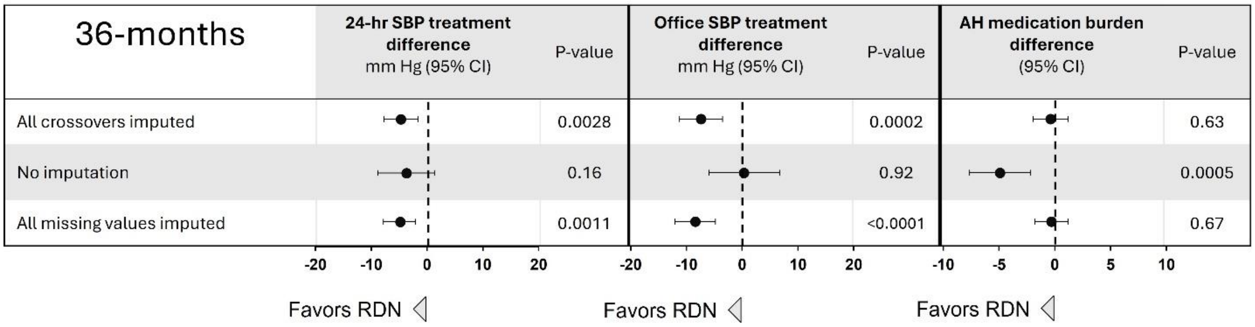

Differences in SBP were ANCOVA adjusted for baseline values. SBP, systolic BP; AH, antihypertensive.

Figure S4. Systolic BP changes between RDN and sham control groups after imputation for all missing measures

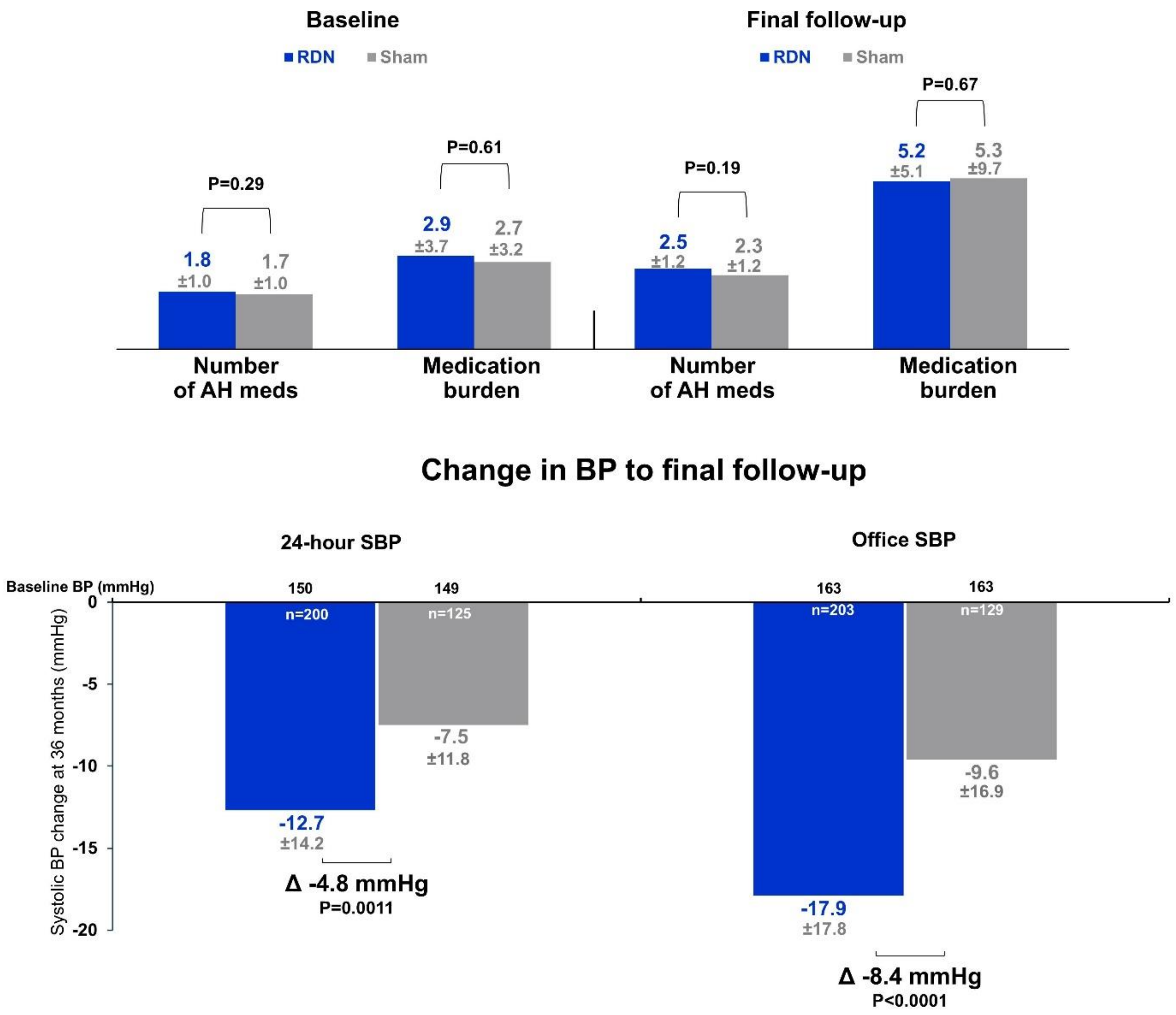

The number of antihypertensive medications and medication burden are plotted (top) at baseline and 36 months after imputing for all missing values. Twenty-four hour ambulatory and office systolic blood pressure changes from baseline to final available follow-up are plotted (below) for RDN and sham control groups.

Figure S5. Blood pressure treatment differences in predefined subgroups at 36 months

Subgroup		RDN N	Sham N	24-h ambulatory systolic BP adjusted treatment difference mmHg (95% CI)
**Sex**	Male	131	86	
	Female	24	25	
**Age**	< 65	134	95	
	≥ 65	21	16	
**BMI**	Tertile 1 (<28.9)	58	32	
	Tertile 2 (28.9 to 33.1)	51	40	
	Tertile 3 (≥33.1)	46	39	
**Type 2 Diabetes**	Yes	16	16	
	No	139	96	
**Geography**	U.S.	59	52	
	Outside U.S.	96	59	
**Race (within U.S.)**	Black American	19	16	
	Non-Black American	40	36	
**Baseline 24-Hour SBP by Tertiles**	1: < 145	49	36	
	2: 145 – 152	51	45	
	3: ≥ 152	55	30	

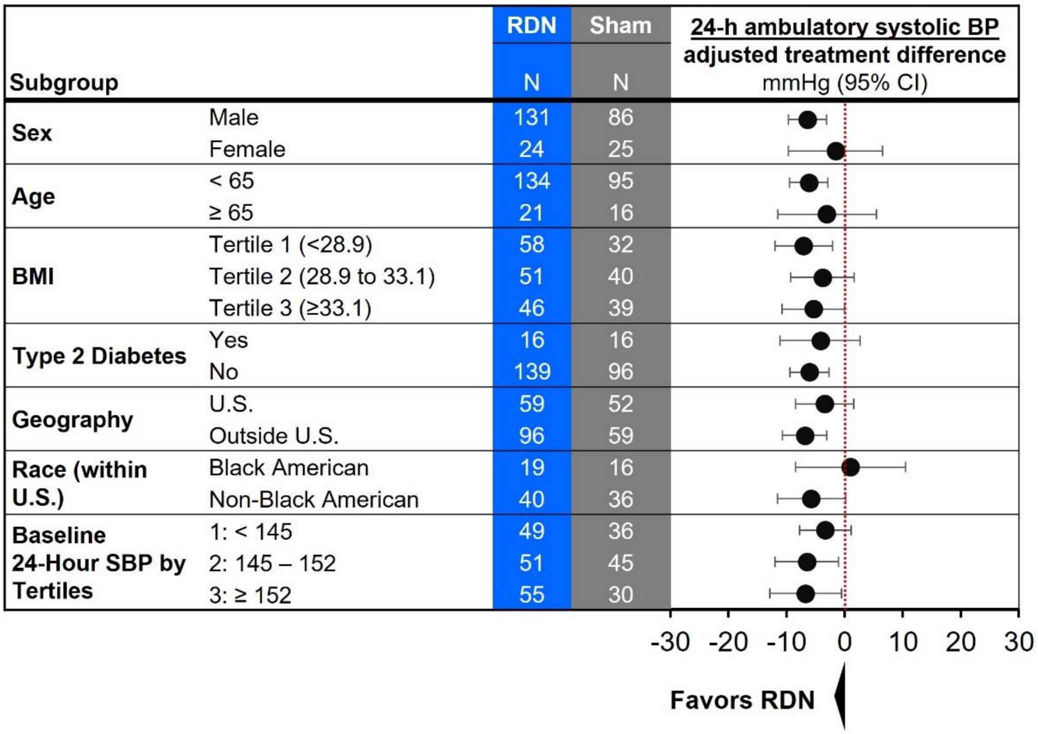

Subgroup		RDN N	Sham N	Office systolic BP adjusted treatment difference mmHg (95% CI)
**Sex**	Male	144	90	
	Female	31	26	
**Age**	< 65	149	100	
	≥ 65	26	16	
**BMI**	Tertile 1 (<28.9)	69	33	
	Tertile 2 (28.9 to 33.1)	55	42	
	Tertile 3 (≥33.1)	51	41	
**Type 2 Diabetes**	Yes	19	18	
	No	156	98	
**Geography**	U.S.	71	56	
	Outside U.S.	104	60	
**Race (within U.S.)**	Black American	24	20	
	Non-Black American	47	36	
**Baseline Office SBP by Tertiles**	1: < 159	62	41	
	2: 159 – 166	55	33	
	3: ≥ 166	58	42	

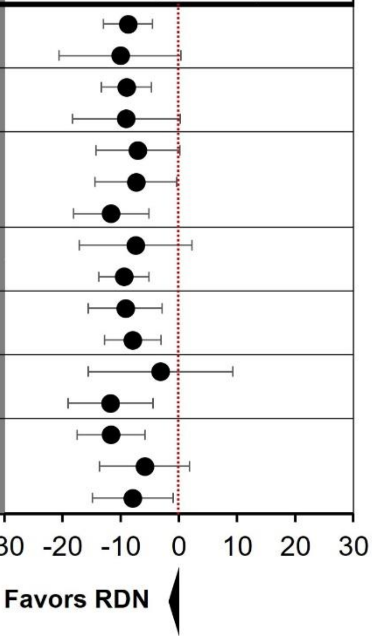

Treatment differences within subgroups were ANCOVA adjusted for baseline BP. BMI indicates body mass index; SBP, systolic BP.

Figure S6. Antihypertensive medications and systolic BP changes among patients with resistant hypertension

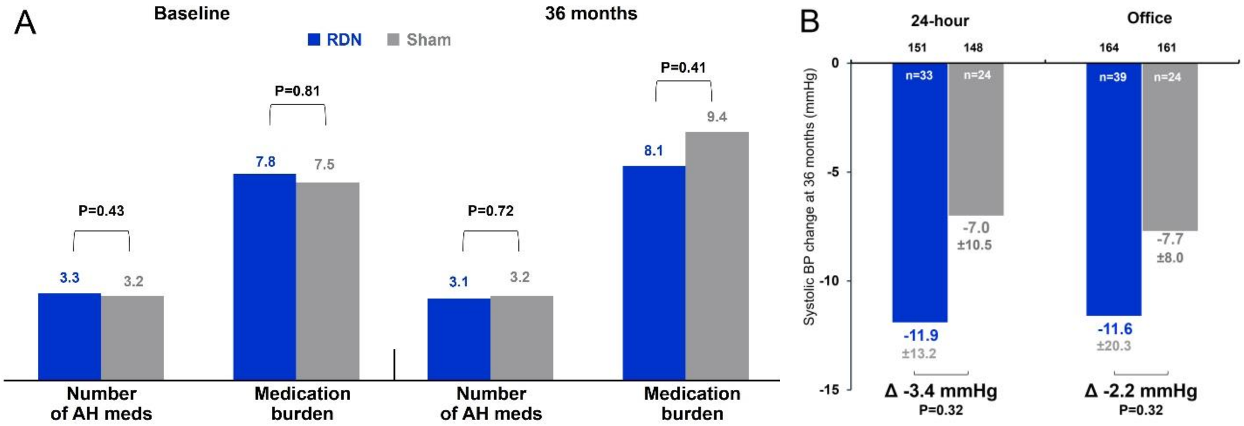

The number of antihypertensive medications and medication burden among patients with resistant hypertension (taking 3 or more AH medications) are plotted **A** at baseline and 36 months. Renal denervation (RDN) group is shown in blue, and the sham control group is shown in gray. The plotted data are based on drug testing information if available, otherwise prescribed information is used. In **B**, the 24-hour ambulatory and office-visit systolic blood pressure (BP) changes at 36 months from baseline ± the standard deviation are plotted for RDN and sham control groups. Comparisons of medication intake and BP changes were conducted by analysis of covariance. For blood pressure measures, comparisons were adjusted for baseline BP. Values for sham control patients who crossed over before 36-month follow-up are imputed by LOCF.
